# Neuropsychological aspects of impulse control disorders in Parkinson’s disease

**DOI:** 10.3389/fnagi.2026.1768251

**Published:** 2026-03-09

**Authors:** Livia Scanferla, Atbin Djamshidian

**Affiliations:** Department of Neurology, Medical University of Innsbruck, Innsbruck, Austria

**Keywords:** binge eating, compulsive sexual behavior, compulsive shopping, dopamine dysregulation syndrome, gambling disorder, neuropsychology, behavior, Parkinson’s disease

## Abstract

Impulse control disorders (ICDs), such as excessive gambling, compulsive sexual behavior, binge eating, compulsive shopping as well as punding, and the dopamine dysregulation syndrome, may arise as a debilitating neuropsychiatric complication in Parkinson’s disease (PD). Although the pathophysiology is not fully understood, it likely involves mesolimbic dopaminergic overstimulation combined with disease-related vulnerabilities in reward, motivation, and inhibitory control networks. This narrative review summarizes evidence on the neuropsychological, affective, and behavioral traits associated with ICDs in PD, with a particular focus on epidemiology/clinical manifestations, neurobiological and pharmacological mechanisms, as well as prevention and management strategies. ICDs can affect up to 40% of PD patients and are strongly associated with dopamine agonist exposure, younger age of onset, premorbid personality traits, and neuropsychiatric comorbidities. Neuropsychological findings reveal abnormalities in several domains, including reflection impulsivity, temporal discounting, novelty seeking, risk processing, and inhibitory control, while mood disorders, sleep dysfunction, apathy, and anxiety further influence vulnerability and worsen behavioral dysregulation. Although general awareness for development of ICDs has been raised, they still represent a significant burden for patients and their family members and are a predictor of functional decline and lower quality of life. Management includes dopamine agonist withdrawal whenever possible, the cessation of fast acting dopaminergic agents and treatment of neuropsychiatric comorbidities. In selected cases, deep brain stimulation or continuous dopaminergic delivery should be considered, particularly in those experiencing persistent worsening of motor symptoms despite appropriate adjustment of dopaminergic medication.

## 1 Introduction

Idiopathic Parkinson’s disease (PD) is the fastest-growing neurodegenerative disorder worldwide with a 2.4 increase from 1990 to 2016 (Kalia and Lang, 2015; Feigin et al., 2019). The disease is more common in men and typically manifests in older adults, with a median age of onset at 60 years (Cheng et al., 2010; Dorsey et al., 2018).

Pathologically, PD is defined by a progressive degeneration of dopaminergic neurons primarily within the substantia nigra pars compacta (SNpc), especially within the ventrolateral tier where neurons project to the dorsal putamen of the striatum (Ramesh and Arachchige, 2023). By contrast, the ventral striatum, which receives dopaminergic input from the ventral tegmental area (VTA), remains relatively preserved at disease onset (MacDonald and Monchi, 2011).

The classical motor phenotype of PD includes bradykinesia, rigidity and tremor (Moustafa et al., 2016). In addition, gait disturbances and postural instability become a later feature of the disease (Bloem et al., 2021), accompanied by a shortened stride length and a progressively stooped posture (Talaat et al., 2021). Postural instability further contributes to mobility impairment (Skidmore et al., 2022).

Apart from motor symptoms, non-motor symptoms, including cognitive impairment, sleep and mood disorders, and a spectrum of behavioral changes, significantly contribute to a reduced quality of life for patients as well as their family members (Hermanowicz et al., 2019; Balestrino and Schapira, 2020; Bloem et al., 2021).

Among these behavioral changes, impulse control disorders (ICDs) have emerged as a complex and often under-diagnosed phenomenon ( Ávila et al., 2011).

According to the ICD-11, ICDs are defined as mental disorders characterized by a failure to resist an impulse, drive, or temptation to act in a way that is potentially harmful. They are typically preceded by mounting tension and followed by feelings of pleasure, gratification, or relief (Evans et al., 2019; World Health Organization, 2022). The DSM-5 categorizes ICDs as oppositional defiant disorder, intermittent explosive disorder, conduct disorder, kleptomania, and pyromania. It differentiates between nine distinct forms of substance addiction, including alcohol, cannabis, opioids, and stimulants (American Psychiatric Association, 2013). Within PD, ICDs include gambling disorder, compulsive sexual disorder, compulsive buying, and binge eating, as well as related behaviors such as punding (repetitive, purposeless activities), hobbyism, and the compulsive overuse of dopaminergic medication, also known as dopamine dysregulation syndrome (Evans et al., 2019).

Reported prevalence rates of ICDs in PD differ widely, with studies reporting 15% (Weintraub et al., 2022), to 28% (Antonini et al., 2017) and up to 40% (Cossu et al., 2018). Moreover, ICDs seem to be lower in Asia compared to Western countries, probably due to cultural, socio-economical and political differences (Parra-Díaz et al., 2021).

Notably, the prevalence rises substantially with disease duration, reaching a cumulative 5-year incidence of 46%, underscoring the considerable variability in reported rates and the influence of methodological and clinical factors (Corvol et al., 2018).

In fact, several instruments have been developed to identify and assess ICDs in PD. The Questionnaire for Impulsive-Compulsive Disorders in Parkinson’s Disease (QUIP) is a widely used screening tool. It uses a dichotomous response format and is divided in three sections: common ICDs, hobbyism/punding/walkabout, and compulsive medication use (Weintraub et al., 2009). However, it does not capture symptom severity. To address this, the QUIP-Rating Scale (QUIP-RS) was introduced. It employs a Likert scale that allows patients to rate the severity of ICD symptoms according to frequency, assessing both presence and severity of ICDs and therefore increasing clinical utility (Weintraub et al., 2012).

The main objective of this review was to summarize the neuropsychological, affective, and behavioral traits associated with the development of ICDs in PD. More specifically, we aim to address the following questions:

(1) Which cognitive, affective, and behavioral traits are most consistently associated with the development and expression of ICDs in PD? (2) What is the reported epidemiology of ICDs in PD, and how do clinical manifestations vary across ICD subtypes and disease stages? (3) Which neurobiological and pharmacological mechanisms—especially within rewards and inhibitory-control circuits—contribute to heightened vulnerability? (4) Is there evidence supporting current strategies for prevention, screening, and clinical management?

Several recent reviews (Carbone and Djamshidian, 2024) have summarized ICDs in PD with a particular focus on epidemiology, risk factors, and management. In contrast, in this present review we focus on the neuropsychological aspects and organize the literature around specific cognitive and affective vulnerability constructs (rather than a medication-side-effect narrative alone). We further translate these constructs into a clinically useful framework for screening, phenotyping and interpretation, highlighting why findings can appear mixed across tasks and ICD subtypes and how this can be resolved with future subtype-sensitive assessments.

As a narrative review, we integrate both seminal work and more recent studies that refine epidemiology, mechanisms, and management. Where relevant, we explicitly highlight recent advances within each subtopic to address changes that may have occurred over the past years.

## 2 Importance and impact of ICDs

### 2.1 Social isolation and quality of life

ICDs emerge as independent predictors of decreased quality of life, especially when it comes to emotional wellbeing. They are correlated with an increased disability, emphasizing their impact on functional impairment (Phu et al., 2014). Psychosocial factors can worsen these outcomes. PD patients frequently retreat from social connections due to stigma and often choose solitary, low-engagement activities (Delaney et al., 2012). Such isolation, along with the difficulties of managing a chronic disease, may enhance vulnerability to maladaptive behaviors, as boredom and loneliness are known triggers for pathological gambling in the general population (Grant and Kim, 2001). PD patients with ICDs have greater levels of social introversion, which is linked to decreased self-esteem (Baumann-Vogel et al., 2015).

The clinical consequences of ICDs may be profound, as they can have a significant impact on patients’ lives and often cause long-lasting psychosocial and financial harm (Phu et al., 2014; Antonini et al., 2017). Affected patients may experience relationship problems, including divorce, bankruptcy, incarceration, or even attempt suicide (Lim et al., 2008; Baig et al., 2019). Despite similar motor severity, PD patients with ICDs exhibit greater functional impairment in daily living (Voon et al., 2011a) and experience more frequent non-motor symptoms such as urinary and cardiovascular dysfunction (Jesús et al., 2020) and poorer sleep quality (Antonini et al., 2017).

### 2.2 Loss of insight and caregiver burden

Impaired awareness and limited reporting of ICDs is a well-known problem. Many patients do not consider their ICD symptoms to be problematic (Leeman et al., 2012). Moreover, the often humiliating or stigmatizing aspects of these addictive behaviors can lead to intentional hiding of information due to shame or denial. Discrepancies between patient self-reports and reports from spouses or family members are also frequently observed (Mestre et al., 2013). Furthermore, patients and family members may be unaware that ICDs are associated with dopaminergic medication, and therefore do not report these symptoms (Vilas et al., 2012).

Caregiver burden describes the physical, emotional, and economic challenges associated with caring for people with chronic and disabling diseases, such as PD. The resulting physical and psychological strain of caregiving reduces opportunities for self-care. Additionally, caregivers are often forced to limit their personal and social activities. A decreased social network, and the subsequent social isolation, are perceived as a critical loss of support. All this increases the risk of comorbidities (Roland et al., 2010; Leroi et al., 2013; Aamodt et al., 2024).

Caregivers of PD patients with behavioral problems, such as ICDs, report even higher levels of burden and heightened distress (Leroi et al., 2013; Mosley et al., 2017). Non-motor symptoms consistently have a bigger impact on caregiver burden than motor symptoms because they aggravate impairment, increase the need for supervision, and therefore strain the relationship between patient and caregiver. ICDs often result in severe financial, social, and legal consequences. Moreover, secrecy surrounding compulsive behaviors, a lack of insight in patients, and refusal of interventions add additional stress in interpersonal relationships. Perception that the patient is behaving willfully can lead to resentment among caregivers (Chahine et al., 2021). Studies show that the neuropsychiatric burden and the caregiver’s own health status are strong predictors of the overall perceived burden (Johnson et al., 2023). Qualitative research also suggests that changes in interpersonal dynamics, such as the shift from an equal partnership to a dependency on caregivers, contribute significantly to the subjective experience of burden (Geerlings et al., 2023).

## 3 Most common addictive behaviors

### 3.1 Compulsive sexual disorder

Compulsive sexual behavior (CSB) is defined as a persistent pattern of inability to control strong, repetitive sexual urges that result in repetitive sexual behavior. It is often under-diagnosed and can significantly impair one’s ability to function in social, familial, educational, professional, or other crucial domains (World Health Organization, 2022). CSB is considered one of the most frequent ICDs in PD, with evidence suggesting that it is more prevalent in men and tends to emerge earlier than other ICD subtypes. The behavior is linked to a younger age at PD onset and a higher levodopa dose compared to other forms of ICD. Patients with CSB are also more likely to develop multiple ICDs (Codling et al., 2015; Barbosa et al., 2018). The prevalence rate in PD is estimated to be around 3%–3.5% (Weintraub et al., 2010; Griffin et al., 2021). However, reported prevalence rates vary widely across studies, ranging from 1.9% to 22.8%. This heterogeneity is attributed to methodological differences caused by the lack of a validated screening instrument for CSB and the frequent reliance on the QUIP for the assessment of CSB (Tayim et al., 2024).

### 3.2 Gambling disorder

Gambling disorder (GD) is defined as a persistent and recurrent maladaptive pattern of gambling behavior that leads to significant impairment or distress (American Psychiatric Association, 2013). It is characterized by impulsive actions that disturb patients’ lives and those of their families, with traits like cognitive salience, conflict with other life domains, temporary euphoria or relief, tolerance, withdrawal, and relapse. Prevalence rates in PD range from 2.2% to 7% (Culicetto et al., 2024), with male gender, younger age at PD onset, and a personal or family history of gambling, alcohol, or substance abuse identified as risk factors (Santangelo et al., 2013; Culicetto et al., 2024). Cross-cultural studies suggest that the prevalence is generally higher in Caucasian than in Asian populations. The difference may be linked to cultural components as well as methodical issues, like the reliance on self-administered surveys, as the patient often lack awareness or conceal their behavior due to shame and denial (Santangelo et al., 2013). GD is also associated with worse mental health both before and after diagnosis (Wolfschlag et al., 2025). Importantly, dopamine agonist therapy has been strongly linked to GD and other ICDs. It is therefore crucial to counsel patients about these risks beforehand (Djamshidian et al., 2011a).

### 3.3 Dopamine dysregulation syndrome

Dopamine dysregulation syndrome (DDS) is a neuropsychiatric condition in which PD patients develop addiction to their dopaminergic medication. Affected individuals self-administer dosages, or continually request increase from doctors, that are higher than what is needed for motor symptom control, often despite side effects such as debilitating drug-induced dyskinesias (Liang et al., 2023). Proposed diagnostic criteria for DDS emphasize the excessive and increasing use of dopaminergic therapy despite severe side effects. Medication hoarding and obtaining extra dopaminergic drugs are also commonly observed (O’Sullivan et al., 2009). Patients with DDS frequently experience significant social or occupational impairment and may exhibit withdrawal symptoms after dose reduction (Giovannoni et al., 2000). DDS prevalence rates vary significantly amongst research, ranging from 2.2% to 10.4% (Liang et al., 2023). Men and patients with an early onset of PD are more likely to develop DDS (Warren et al., 2017). Additionally, patients with ICD-related symptoms such as hypersexuality, obsessive eating, and compulsive shopping appear to be at higher risk (Liang et al., 2023).

### 3.4 Compulsive shopping

Compulsive shopping is characterized by repetitive, impulsive, and excessive purchasing of items that are not needed and often result in financial stress. Affected individuals lack control over the behavior and have an overwhelming urge to purchase goods (Cho et al., 2008). In PD, compulsive shopping appears to occur more frequently in female patients (Weintraub et al., 2015) and is more common in patients with baseline depression, as well as younger age (Marković et al., 2020). Estimates of prevalence vary considerably, ranging from small studies reporting a frequency of 0.4%, to larger studies reporting a prevalence of 5.7% (Evans et al., 2009).

### 3.5 Binge eating

Binge eating occurs when an individual consumes an excessive amount of food, exceeding what is required to ease hunger (De Chazeron et al., 2019). Binge eating affects about 2% of the general population in the United States. In the DOMINION study from 46 movement disorder clinics in the United States and Canada, binge-eating disorder affected 4.3% of PD patients with ICDs (Weintraub et al., 2015). Women are more likely to suffer from it (Vela et al., 2016; De Chazeron et al., 2021), although results have been inconsistent, with other studies indicating that men are more prone to eating disorders (Bhattacharjee, 2018). Impulsivity, notably higher scores on attentional and non-planning variables, as well as mania have been linked to it and its severity (Zahodne et al., 2011; De Chazeron et al., 2021). Weight change is not a reliable warning sign for this type of ICD in PD. The connected weight gain is initially perceived positively by the patient, or is compensated by pre-diagnostic weight loss. Additionally, binge eating is not necessarily associated with weight gain or obesity (De Chazeron et al., 2019), which further requires physicians’ vigilance to detect and screen for this addiction.

### 3.6 Punding

Punding is a complex phenomenon defined by aimless and non-goal-directed behavior that can last for extended periods, frequently at the expense of other everyday responsibilities. Patients demonstrate an unusual obsession with objects, such as collecting, sorting, or disassembling and reassembling items (Spencer et al., 2011). Punding is not primarily motivated by rewards, pleasure, or obsessive-compulsive tendencies. Contrastingly, it is often perceived as unrewarding and disruptive. However, interruptions during the activity cause impatience, anxiety, or annoyance (Rajalingam and Fasano, 2023). Clinically, punding activities often result in sleep deprivation, strained relationships, and social disengagement (O’Sullivan et al., 2007; Evans et al., 2019). The most common etiology in PD is exposure to high doses of dopaminergic treatment. Epidemiological studies show a variable prevalence, ranging from 1.4% in unselected PD cohorts (Miyasaki et al., 2007) to 14% in patients treated with higher doses of levodopa (Evans et al., 2004). While some studies show greater prevalence among men (Rajalingam and Fasano, 2023), others have found no significant demographic or disease-related determinants (Miyasaki et al., 2007). Overall, punding appears to be underreported, most likely due to low awareness and patients’ reluctance to disclose socially inappropriate behaviors (O’Sullivan et al., 2007). Importantly, patients may lack insight into the pathological nature of their behaviors, with caregivers being often the first to recognize the negative psychosocial impact (O’Sullivan et al., 2007).

### 3.7 Miscellaneous addictive behaviors

Reckless generosity refers to inappropriate, uncontrolled giving that can disrupt the financial stability and social relationships of patients (O’Sullivan et al., 2010b). Although generosity is usually positive and seen as the willingness to help others beyond what is expected, excessive helping can become maladaptive and socially inappropriate (Amstutz et al., 2021). Research suggests that this behavior may come from impaired decision-making mechanisms, similar to behavior observed in other ICDs. Dopaminergic drugs can alter rewards processing by reducing sensitivity to negative outcomes and increasing activation in mesolimbic rewards circuits associated with charitable giving, including the ventral striatum and subgenual cingulate. This may contribute to dysfunctional social rewards processing in PD patients with ICDs (O’Sullivan et al., 2010b; Ponsi et al., 2021).

Excessive hoarding is the pathological collecting of items while struggling to discard them, resulting in extreme clutter. Approximately 12% of PD patients display excessive hoarding (O’Sullivan et al., 2010a).

Deficits in social cognition, including theory of mind and moral decision-making, have been documented in PD. Moral behavior is regulated by networks in cortical and subcortical regions that are often affected by PD pathology, even in early phases (Santens et al., 2018). Cohen and Millet (2020) described a patient who, after starting ropinirole, was arrested for maiming and killing dozens of cats. The behavior ended after he stopped taking the medication. Micheli et al. (2015) reported a patient on increasing doses of pramipexole who adopted cats but then felt an uncontrollable urge to kill them, while expressing distress and confusion about his actions.

Additional evidence of behavioral dysregulation on dopaminergic therapy includes cases in which PD patients developed compulsive crack cocaine use after DA treatment (Friedman and Chang, 2013).

Impulse control disorders can occur in isolation, however, in about half of cases, patients may present with more than one addiction. Most ICDs may arise from idiosyncratic hobbies or behaviors and can increase in severity, if not treated accordingly. Furthermore, research suggests that ICDs may be categorized into intrinsic, extrinsic and learned rewards. Compulsive eating and hypersexuality involve intrinsic rewards, whereas gambling and compulsive shopping rely on socially learned, extrinsic rewards, implying different underlying neurobiological mechanisms. Neuroimaging studies reporting cases of ICDs emerging after subthalamic deep brain stimulation suggest that different ICDs rely on distinct neural circuits depending on whether the rewards is primary or secondary (Marques and Lewis, 2025).

Recent population-level registry evidence further supports dopaminergic exposure and psychiatric comorbidity as key predictors for ICD s in PD (Wolfschlag et al., 2025). At the same time, recent update reviews continue to emphasize substantial heterogeneity in reported prevalence, reflecting differences in screening instruments and underreporting due to shame, denial or limited insight (Marques and Lewis, 2025).

## 4 Pathophysiology of ICDs in PD

The pathophysiology of ICDs in PD is still incompletely understood. While dopaminergic degeneration of the nigrostriatal system causes the classical motor impairments, ICDs appear to emerge from a combination of disease-related vulnerability and treatment effects. As such, various hypotheses have been proposed on the pathophysiology.

Generally, dopaminergic therapies are given to restore dopamine levels within the dorsal striatum to alleviate motor deficits. However, these treatments can also affect the mesolimbic dopaminergic circuitry, which plays a central role in rewards processing and impulse regulation (Cools et al., 2003). Consequently, in vulnerable patients, dopaminergic medication may inadvertently overstimulate mesolimbic pathways, which has been labeled as “dopamine overdose” hypothesis (Van Der Vegt et al., 2020). This overstimulation of rewards-related circuits can lead to maladaptive behavioral patterns and is thought to contribute to the emergence of ICDs. This theory is supported by a neuropathological study showing that PD patients with ICDs exhibit lower α-synuclein pathology and reduced D3 receptor levels in the nucleus accumbens. This suggests that the ventral striatum remains relatively preserved compared to the more severely affected dorsal striatum, which shows advanced degeneration in both groups (Barbosa et al., 2019a).

Positron emission tomography (PET) studies have added significant contributions to this field. For example, PD patients with DDS had higher L-dopa-induced ventral striatal dopamine release during PET scanning compared to PD controls (Evans et al., 2004). In line with this, PD with various ICDs also had increased ventral striatal dopamine release in response to rewards-related cues (O’Sullivan et al., 2011; Wu et al., 2015). Aligning with these results, the incentive-sensitization theory (IST) was proposed (Robinson and Berridge, 2025). The IST suggests that repeated exposure to addictive drugs can lead to persistent sensitization of mesolimbic dopamine systems in vulnerable individuals. This sensitization increases the motivational salience of drug-related stimuli, resulting in excessive and compulsive “wanting” without an increasing, or even a decreasing, “liking” for the substance. The theory is based on three findings: (1) Repeated drug use causes long-term mesolimbic sensitization, which is characterized by increased dopamine release and related neural adaptations; (2) this sensitization lasts long, even after the drug use is stopped; and (3) dopamine primarily mediates incentive salience or cue-triggered “wanting,” rather than the pleasure of rewards. As a result, drug cues become triggers for outbursts of “wanting,” even if the drug itself is no longer enjoyable (Robinson and Berridge, 2025).

Neuroimaging studies suggest that levodopa-induced dyskinesias (LID) and ICDs have partially overlapping pathophysiological mechanisms. LID are linked to reduced dopamine transporter availability in the putamen, an increased dopamine turnover, and a sensitized dopamine response in the ventral striatum after levodopa administration. ICDs are associated with heightened saliency-network activation in response to rewards cues, and abnormalities in cortico–subthalamic coherence (Voon et al., 2017). Furthermore, patients with parkin mutation, who are predisposed to developing LID, may develop more severe ICDs even on lower levodopa doses (Biundo et al., 2017).

Generally, there is a close relationship between dyskinesias and ICDs. In fact, dyskinesias are significantly more common in people with ICDs, and patients with multiple ICDs have higher dyskinesia scores. Although the exact mechanism remains unknown, it has been hypothesized that dopaminergic therapy may cause neuroplastic changes in the motor loop, causing dyskinesias and changes in the mesocorticolimbic system, such as sensitization (Biundo et al., 2017; Voon et al., 2017; Hinkle et al., 2018). Importantly, therapeutic strategies aimed at minimizing dopaminergic stimulation, such as continuous levodopa/carbidopa intestinal gel or apomorphine infusion, show promising results in reducing both dyskinesias and ICD severity. This supports the hypothesis that intermittent dopaminergic stimulation contributes to their co-occurrence (Carbone and Djamshidian, 2024; Heim and Djamshidian, 2025).

Although dopaminergic dysfunction is central, increasing evidence highlights the contribution of other neurotransmitter systems to the emerge of ICDs, especially those involving serotonin and noradrenaline (Delaville et al., 2011). Alterations in serotonergic transmission have been shown to impair inhibitory control and decision-making processes (Rogers, 2011). Dysregulation of serotonergic function has also been linked to motivational deficits in PD (Nobis et al., 2023). Although mesolimbic dopaminergic overstimulation remains a central hypothesis on the pathophysiology of ICDs, recent evidence extends beyond dopamine: double-tracer PET findings implicate serotonergic alterations associated with ICD status and severity, supporting a broader neurochemical model that may help explain inter-individual and subtype heterogeneity (Prange et al., 2025). Noradrenergic mechanisms have also been implicated, with studies showing that pharmacological therapies using atomoxetine, a selective noradrenaline reuptake inhibitor, can improve inhibitory control in PD patients without psychiatric comorbidities (Ye et al., 2015).

## 5 Risk factors

Dopamine agonists (DAs) represent the primary pharmacological risk factor for developing ICDs in PD, since they act on D2- and D3-type receptors that are highly expressed within the mesocorticolimbic system. DAs bind to D2-type receptors (D2, D3, D4) over D1-type receptors, which can overstimulate D3-mediated rewards pathways and excessively disinhibit D2-mediated indirect pathway signaling (Stacy, 2009; Kelly et al., 2020). This receptor selectivity contributes to ICD vulnerability, with pramipexole showing the highest association, followed by ropinirole, rotigotine, and pergolide. Apomorphine and bromocriptine are linked to lower risk (Seeman, 2015). In addition, pulsatile dopaminergic therapy can sensitize D3 receptors within mesocorticolimbic circuits, further facilitating behavioral addictions linked to D2/3 receptor activation (Béreau et al., 2019).

Differences between DA formulations further influence risk. Oral DAs are associated with a higher incidence of ICDs than transdermal formulations (Garcia-Ruiz et al., 2014; Rizos et al., 2016; Vitale et al., 2019). Furthermore, combined DA and levodopa medication can increase the risk of developing ICDs (Weintraub et al., 2010, 2015; Weiss and Marsh, 2012; Cao et al., 2022).

In contrast, ICDs are less common with levodopa monotherapy. Studies show that the prevalence of ICDs in PD patients receiving levodopa monotherapy is only slightly higher than in the general population. Exceptions occur with unusually high doses of levodopa (Weiss and Marsh, 2012; Bastiaens et al., 2013). Importantly, ICD symptoms often improve after switching from DAs to slow-release levodopa/carbidopa (Bastiaens et al., 2013; Lee et al., 2019). ICDs are also less frequently associated with amantadine and MAO-B inhibitors such as rasagiline (Grassi et al., 2021).

Newly diagnosed, drug-naïve PD patients show no increased rates of ICDs, or compulsive behaviors compared to healthy controls (Antonini et al., 2011; Lee and Jeon, 2014; Zhu et al., 2023). However, it is important to note that the majority of patients exposed to medications will never develop ICD, indicating a multifactorial etiology (Marques et al., 2018).

Sleep disturbances represent an additional and often under-recognized risk factor. Large cohort studies show that daytime sleepiness and REM sleep behavior disorder (RBD) independently predict ICD, emphasizing the necessity of screening for, and treating comorbid sleep problems (Kim et al., 2014; Ye et al., 2024). Along with dopaminergic adjustment, improving night-time sleep has emerged as an important additional strategy, as excessive daytime sleepiness, poor sleep efficiency, and sleep fragmentation are linked to an increased risk and severity of ICDs (Scullin et al., 2013; Tang et al., 2024). Non-medication treatment for insomnia in PD patients include cognitive-behavioral therapy for insomnia, light therapy, exercise, or brain stimulation techniques (Duan et al., 2025).

**FIGURE 1:**
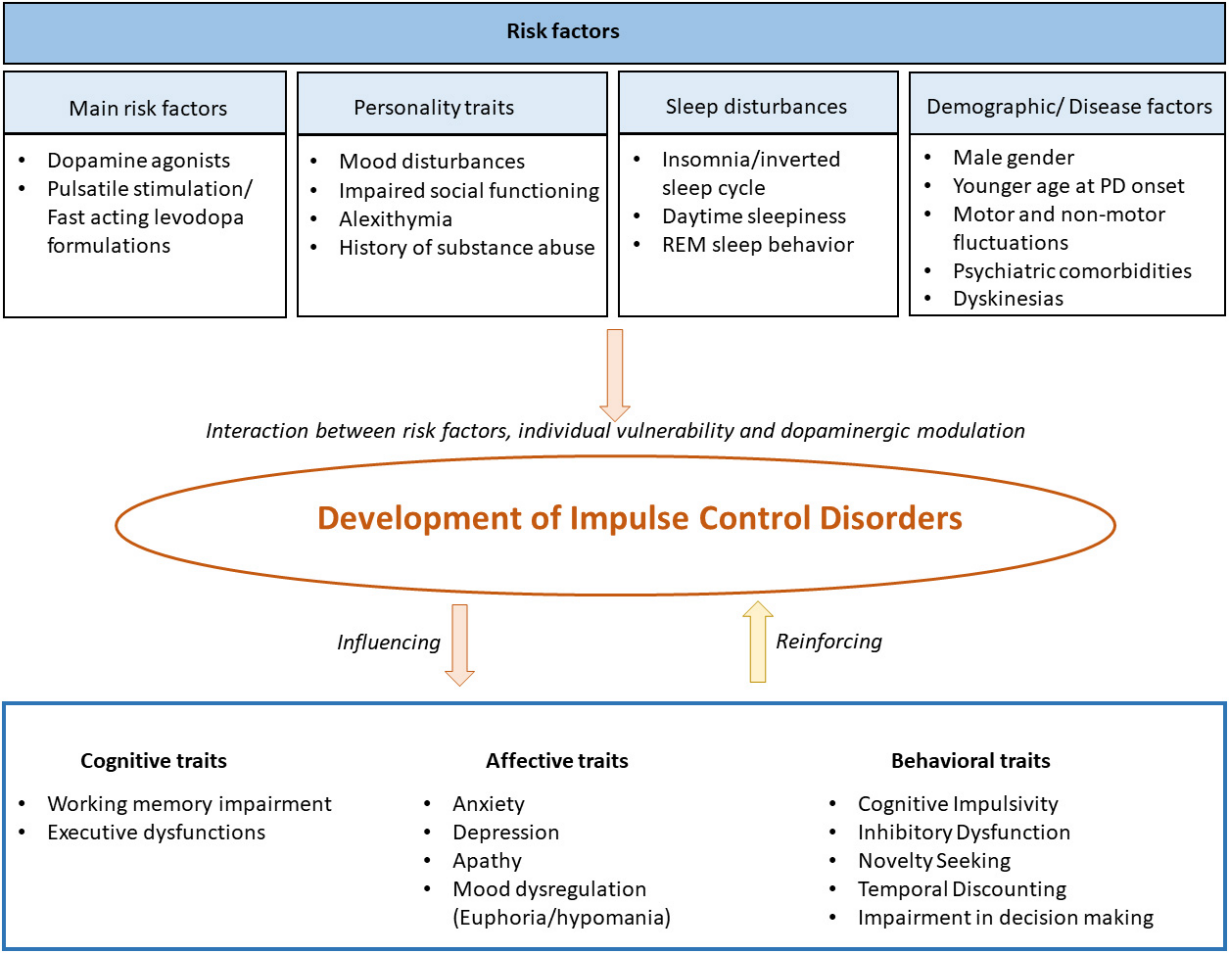
Risk factors for impulse control disorders in Parkinson’s disease.

Beyond pharmacological and sleep-related factors, other non-pharmacological risk factors contribute to vulnerability. Male gender, younger age at PD onset, longer disease duration, and psychiatric comorbidities, such as depression, can increase ICD risk (Pontone et al., 2006; Joutsa et al., 2012; Marín-Lahoz et al., 2019). Other neuropsychiatric symptoms include anxiety (Waskowiak et al., 2022), neuroticism (Callesen et al., 2014), apathy (Baig et al., 2019), and a personal or family history of substance abuse or impulse-related behaviors (Ceravolo et al., 2010; Cilia and Van Eimeren, 2011; Latella et al., 2019). Recent studies show that DA use interacts with psychiatric symptoms, influencing ICD risk. Patients with higher depressive symptoms taking DAs were more likely to develop an ICD, suggesting that depression related difficulties in impulse regulation may heighten vulnerability to DA induced impulsivity (Whooley et al., 2025). Personality traits such as higher novelty seeking and impaired planning have also been associated with ICD risk (Isaias et al., 2008; Carbone and Djamshidian, 2024). Genetic variations affecting dopamine receptor expression and function may further influence susceptibility (Béreau et al., 2019). The risk factors for ICDs are summarized in Figure 1.

## 6 Neuropsychological findings

A recent review provides a detailed synthesis of impulsive action versus impulsive choice in PD across medication states (Warden et al., 2025). Here, we build on and complement that work by focusing specifically on PD + ICDs and by integrating behavioral constructs with ICD subtype/phenotype heterogeneity and clinical implications for screening and interpretation.

### 6.1 Personality traits

Personality traits and behavioral aspects have increasingly been recognized as important contributors to the onset and expression of ICDs in PD. While the role of dopaminergic therapies in triggering ICDs has been well-established, personality factors appear to shape individual vulnerability and influence the way such behaviors manifest.

Maladaptive personality traits have frequently been associated to PD patients with ICDs. ICDs have also been linked to a variety of psychiatric symptoms, including psychoticism and interpersonal sensitivity. They also emerge as a strong predictor of psychoticism, surpassing even disease duration and dopaminergic medication (Jaakkola et al., 2014). Neuroticism appears to be a particular risk factor, as higher levels of neuroticism increase the chance of ICD symptoms and medication addiction (Callesen et al., 2014). Additionally, PD patients with ICDs exhibit significant irregularities in social functioning, such as alienation and apathy toward their social environment, which are increased by comorbid cognitive and executive deficiencies (Farnikova et al., 2012). More recent studies extended earlier findings by identifying a more specific maladaptive trait profile in ICD patients. They reported elevated levels of neuroticism, perfectionism, introversion, and inflexibility related with obsessive-compulsive personality disorder (Kapsomenakis et al., 2023).

Furthermore, alexithymia is about twice as high in PD patients as in the general population, with the frequency ranging from 21% to 23.8% in both medicated and newly diagnosed, untreated patients (Goerlich-Dobre et al., 2014; Assogna et al., 2016). Alexithymia is a personality trait characterized by difficulty identifying and describing emotions, as well as limited introspection (Goerlich-Dobre et al., 2014). It affects around 10% of the general population and is considered a risk factor for the development of ICDs, including pathological gambling, compulsive buying, and substance abuse. However, recent evidence suggests that it is unrelated to motor symptoms, dopaminergic therapy, or symptom laterality (Culicetto et al., 2024).

### 6.2 Cognitive functions

The impact of ICDs on cognition in PD patients has been extensively studied, revealing subtle changes that may represent vulnerability.

#### 6.2.1 Working memory

PD patients with ICDs have poorer working memory (WM) performance than both controls and PD patients without ICDs (Djamshidian et al., 2010). Structural findings further support this, showing cortical thinning in the middle frontal gyrus in ICD patients, a region crucial for WM and strategic decision-making (Gu et al., 2022). Additional studies reported WM deficits in both groups (Leroi et al., 2013). However, a longitudinal analysis revealed a significantly slower progression of cognitive decline in patients with ICDs over a 3.5-year follow-up, especially in domains associated with frontal lobe functioning such as WM, presenting a more complex picture (Siri et al., 2015).

#### 6.2.2 Executive functions

Several studies using cognitive screening tools did not report significant differences between PD patients with ICDs and those without. The cognitive state of PD patients is not associated with ICD presence or severity, as patients typically demonstrate cognitive performance comparable to non-ICD controls of similar disease duration (Martini et al., 2019). For instance, multiple investigations employing the Montreal Cognitive Assessment (MoCA), Frontal Assessment Battery (FAB), or the Mini Mental State Examination (MMSE) failed to reveal between-group differences (Fründt et al., 2022; Contin et al., 2023; Meng et al., 2023; Erkoc Ataoglu et al., 2024).

Beyond these global measures, more detailed assessments of executive function have produced mixed findings. The Tower of London task, which assesses planning and problem solving, showed no significant group differences in accuracy or execution time. However, PD patients with ICDs exhibited longer initiation times, suggesting subtle impairments in cognitive goal-directed planning or preparation, but this effect was only marginally significant (Hlavatá et al., 2020). PD patients with ICDs on medication did not display impairments in cognitive flexibility (e.g., set-shifting, verbal fluency), whereas patients without ICDs were impaired compared to healthy controls (HCs) (Leroi et al., 2013). Conversely, impaired abstract reasoning, reduced cognitive flexibility, and visuospatial deficits seem to be more prominent in ICD patients (Santangelo et al., 2017).

In PD patients with diagnosed mild cognitive impairment (MCI), ICDs are linked to younger age at onset, longer disease duration, higher dopaminergic treatment, and attentional impairments (Martini et al., 2019). Importantly, while ICD frequency and severity were similar across PD patients with no cognitive alterations, MCI, or dementia, the cognitive patterns associated with ICDs differed depending on the cognitive status (Martini et al., 2019).

Broad cognitive screens often cannot distinguish PD patients with ICDs from those without (Zhu et al., 2023). In contrast, differences become more apparent when assessment focuses on specific decision-making and impulsivity constructs rather than on global cognition alone. Recent work also underscores the importance of characterizing ICDs in terms of severity and clinically meaningful phenotypes, consistent with growing recognition of heterogeneity across presentations and the need for individualized interpretation (Debove et al., 2024; Marques and Lewis, 2025).

### 6.3 Behavioral aspects

Several studies demonstrate that patients with ICDs score significantly higher on the Barratt Impulsiveness Scale (BIS-11) compared to those without ICDs (Aumann et al., 2020; Contin et al., 2023; Giovannelli et al., 2023). Reinforcing this observation, evidence shows significantly elevated BIS-11 scores across all domains in PD patients with ICD, with the most pronounced differences in impulsivity (Giovannelli et al., 2023).

Impulsivity is broadly defined a tendency to act rapidly in pursuit of potential rewards with little planning, and often without considering the risk of punishment or loss of rewards (Evenden, 1999; Poletti and Bonuccelli, 2013). Current perspectives extend this view to include multifaceted behaviors that have adverse outcomes and involve several aspects of decision making. Within this, two distinct types of impulsivities have been identified: motor impulsivity and cognitive impulsivity. Motor impulsivity, also known as response inhibition, is the disinhibition of prepotent reactions (Robert et al., 2009). Cognitive impulsivity is a more complex process that involves the suppression of previously activated cognitive contents (Antonelli et al., 2011).

#### 6.3.1 Cognitive impulsivity

Cognitive impulsivity is the tendency to make rash, poorly considered decisions and to prioritize immediate rewards above long-term consequences. It is typically linked to altered decision. One widely used paradigm to assess it is the Iowa Gambling Task (IGT), which requires considering immediate rewards against long-term losses (Antonelli et al., 2011). PD patients with pathological gambling consistently show decreased cognitive impulsivity on social behavior assessment and perform poorly on the IGT, choosing disadvantageous options more frequently and failing to adapt strategies over time (Rossi et al., 2010). The beads task, which examines “jumping to conclusions,” is another way to evaluate decision making. Participants guess which of two cups beads are drawn from, weighing the cost of drawing extra beads against the rewards for correct choices. PD patients, both with and without ICDs, pathological gamblers, and substance abusers draw fewer beads and made more illogical decisions than healthy controls. Additionally, PD patients with ICDs resembled substance abusers as they frequently relied on a subjective “feeling” of the right choice (Djamshidian et al., 2012b). On the Stroop task, a response inhibition paradigm in which participants must say the ink color of a word while suppressing saying the word itself, no differences were seen between PD patients with and without ICDs (Averbeck et al., 2014). Other aspects of cognitive impulsivity include risk-taking, which can occur in known probabilistic contexts or in ambiguous situations (Antonelli et al., 2011).

#### 6.3.2 Motor impulsivity

Motor impulsivity refers to the tendency of acting previously learned motor reactions despite signals to inhibit them. It is typically assessed using tasks like the Stop-Signal Reaction Time (SSRT) or Go/No-Go paradigms, which evaluate the speed and accuracy of inhibiting habitual responses (Antonelli et al., 2011). Neurostructural data reveals brain differences between PD patients with and without ICDs, supporting the significance of sensorimotor basal ganglia networks in impulsivity. Larger putamen volume and smaller Globus Pallidus externa (GPe) volume were associated with higher impulsive behavior (Ruitenberg et al., 2018). In PD, SSRT is typically prolonged in patients with moderate to severe illness, regardless of global cognitive impairment or disease severity, showing a vulnerability to halting control in this population (Gauggel et al., 2004). However, results are once more conflicting. Across studies using the BIS-11, motor impulsivity does not reliably differ between patients with ICD and patients without. Nevertheless, only few studies report all subscale scores, so no conclusions can yet be drawn about the specific impulsivity profile in PD (Giovannelli et al., 2023). PD patients with active ICDs frequently demonstrate faster inhibition of initiated motor actions compared to PD patients without ICDs and healthy controls, and less receptivity to acting on strong motor impulses in response conflict tasks (Wylie et al., 2012; Claassen et al., 2015). Choice reaction times and accuracy to “Go” stimuli are similar among PD groups, implying that motor responses are initiated and executed correctly (Claassen et al., 2015). Nevertheless, eye-tracking studies using an anti-saccade task found greater error rates in PD patients with ICDs, even if reaction times and direction errors were not linked with clinical scores, indicating subtle deficits in oculomotor inhibition (Barbosa et al., 2019b).

Patients with diagnosed ICDs often describe a perceived lack of self-control, indicating that they have awareness of changes in their cognition and behavior. Beyond its role as a predictive value for ICD onset, impulsivity has also been associated with a decline in perceived quality of life and, consequently, with poorer overall mental health. This happens mainly due to the psychosocial and practical burden of financial losses, legal difficulties, and strained interpersonal relationships (Jeyadevan et al., 2023).

Importantly, increased impulsivity is not limited to patients with an ICD diagnosis. PD patients, even in the absence of an ICD diagnosis, exhibit elevated impulsivity, particularly in the attention and non-planning domains (Aumann et al., 2020). The findings suggest that PD itself may predispose individuals to greater impulsivity even before the onset of ICDs.

#### 6.3.3 Response inhibition

Behavioral results on response inhibition on patients with PD and ICD are mixed. Bentivoglio et al. (2013) observed a trend toward poorer performance in tasks sensitive to frontal lobe dysfunction, such as the go-no-go subtest of the FAB, although the effect did not reach statistical significance. Impaired response inhibition, reflecting hypo-functionality of the orbitofrontal, anterior cingulate, and medial prefrontal cortices, can contribute to poor impulse control and disinhibition in PD (Palermo et al., 2017). PD patients with ICDs were significantly impaired in the Digit Span test, which assesses working memory and inhibitory control (Djamshidian et al., 2010). Some studies did not find group differences (Hlavatá et al., 2020; Esteban-Peñalba et al., 2021), however, functional correlates linked to proactive and restrained inhibition suggested that PD with ICDs resolved inhibitory demands differently compared to patients with only PD and healthy controls (Esteban-Peñalba et al., 2021).

#### 6.3.4 Novelty seeking

In addition, sensation seeking and novelty orientation have been highlighted as relevant personality factors influencing ICD risk (Terruzzi et al., 2025). Novelty seeking is a personality trait that drives exploratory behavior in response to novel or different stimuli. ICDs in PD patients are often associated with higher measures of novelty seeking, compared to those without an ICD (Voon et al., 2007, Voon et al., 2011b; Poletti and Bonuccelli, 2013). These traits vary depending on the disease stage and responsiveness to dopaminergic therapy. As the disease advances and higher dosages of dopaminergic drugs are necessary to control motor symptoms, novelty-seeking behaviors become more prevalent. This is widely believed to be a direct result of dopaminergic treatment (Paulk and Neilson, 2024).

Some studies reported that PD patients with self-reported ICDs displayed higher levels of sensation seeking and novelty orientation prior to PD diagnosis compared to controls, suggesting that these traits may represent premorbid predisposition (Leplow et al., 2023). However, not all studies reached the same conclusion. In newly diagnosed PD patients, a study observed low novelty-seeking scores (Meira et al., 2022). While some studies indicate that medicated PD patients show lower novelty seeking compared to HCs (Poletti and Bonuccelli, 2013), other findings suggest that PD patients with ICDs were more attracted to novel stimuli, independent of medication status and across different ICD subtypes (Djamshidian et al., 2011b). This apparent discrepancy has been addressed by recent hypotheses suggesting that ICD vulnerability can be caused by a mismatch between low baseline novelty-seeking and treatment-induced increases in novelty-driven behavior, rather than novelty seeking itself (Paulk and Neilson, 2024).

#### 6.3.5 Risk taking

Patients with ICDs display higher impulsivity and irrational decision-making (Santangelo et al., 2017), and have an impaired risk evaluation (Voon et al., 2011a; Warden et al., 2025). This is supported by findings reporting that both PD groups demonstrated risk-taking behavior, however, only PD patients with ICDs showed deficits in subjective rewards estimation, focusing mainly on positive reinforcement while neglecting potential losses (Pineau et al., 2016). In contrast, another study found no differences between PD patients with and without ICDs on the Delay Discounting Task, which measures impulsive decision-making and preference for immediate over delayed rewards (Hlavatá et al., 2020). In another study, PD patients with ICDs showed a non-significant trend toward greater risk-taking compared to PD patients without. However, those with pathological gambling were significantly more risk-prone than PD patients without ICDs, a pattern that can typically be observed in pathological gamblers. Dopaminergic medication increased risk preference in PD patients relative to healthy controls, and a similar trend was observed in PD patients with ICDs (Djamshidian et al., 2010). A meta-analysis found a strong association between DA therapy and heightened financial risk-taking in PD generally. The results were supported by neuroimaging evidence showing that DAs amplify risky decision-making, reduce orbitofrontal value sensitivity, and impair negative feedback processing (Kandylaki et al., 2025).

#### 6.3.6 Learning

Throughout the course of the disease, PD patients face numerous challenges to their rewards-learning systems, like the progressive dopaminergic neuron loss, non-physiological administration of exogenous dopamine and dopamine receptor agonists, and excessive dopamine levels caused by high-dose medications. Together, these factors disturb the normal physiological regulation of dopamine release, resulting in altered learning relative to HCs (Lee and Jeon, 2014). Previous studies found that medicated PD patients had a better positive than negative feedback learning while patients off medication showed an opposite behavior. In line with this, DA therapy in young, never-medicated PD patients, who were free of addictive behaviors, enhanced novelty seeking and rewards learning, had impaired punishment learning, confirming dopamine’s role in rewards-based learning at the expense of punishment sensitivity (Bodi et al., 2009). Short-term learning of inhibitory control has been examined using experimental paradigms aimed to simulate everyday settings. In these, accurate inhibition must be acquired without rewards, and failure to suppress a response is punished. Within-session learning of non-rewarded inhibition in PD patients with ICDs was at chance levels. In contrast, HCs quickly learned behavioral inhibition. Patients with PD but without an ICD demonstrated intermediate impairments but eventually improved across trials. When failures to inhibit were punished, both PD groups had little inhibitory learning, with ICD patients showing almost no inhibitory learning at all (Leplow et al., 2017). Evidence suggests that PD patients might overvalue positive feedback while undervaluing negative feedback, which could lead to compulsive behaviors like gambling. While medicated PD patients without ICDs learn better from positively rewarded stimuli than from negatively rewarded stimuli, PD patients with ICDs appear to exaggerate this bias, with increased sensitivity to rewards and less adaptation to stimulus values based on negative prediction errors (Averbeck et al., 2014; Piray et al., 2014; Carbone and Djamshidian, 2024). Unmedicated PD patients, on the other hand, demonstrate improved learning from negative feedback, demonstrating the modulatory effect of dopaminergic treatment on rewards processing. Overall, the findings indicate that PD patients with ICD struggle to accurately predict the importance of inhibitory responses, especially when correct behavior is not externally rewarded. They are disproportionately influenced by positive outcomes, which may contribute to the development of addictive behaviors.

#### 6.3.7 Temporal discounting

Temporal discounting, also known as delay discounting, refers to the preference for smaller immediate rewards over larger delayed rewards. Tasks typically give hypothetical options (e.g., “Would you prefer 5€ today or 20€ in 6 months?”) or provide incentives over short periods (Averbeck et al., 2014). In PD patients with ICDs, studies show that immediate gratification is often preferred (Housden et al., 2010; Antonelli et al., 2011; Leroi et al., 2013). For instance, PD patients with ICDs exhibit increased temporal discounting for both food and monetary rewards, indicating an over-attribute of motivational value to rewards and difficulty delaying gratification. They also exhibit an exaggerated salience to non-rewarding stimuli (Terenzi et al., 2022). However, PD patients who did not have clinically evident ICDs exhibited impulsive behaviors when making financial decisions, independent of medical status. This may be due to dopaminergic therapy’s long-term effects or a medication-independent change in choice behavior in PD (Milenkova et al., 2011). Patients with hypersexuality also showed reduced discounting for erotic delayed rewards compared to PD and HCs, accepting longer waits to view erotic pictures for a longer period of time (Girard et al., 2019).

#### 6.3.8 Reflection impulsivity

Reflection impulsivity is the insufficient information gathering before making a decision and is commonly seen in patients with ICDs. The beads task is typically used for assessment, in which participants decide whether to draw additional evidence before selecting between two containers with different bead-color proportions. Across multiple variants of the task, PD patients with ICDs consistently gather less information and make earlier, often irrational decisions, a pattern also seen in substance-use disorders and pathological gambling (Djamshidian et al., 2013; Potenza, 2014; Voon, 2017). DAs worsen this tendency, increasing reflection impulsivity relative to levodopa or deep brain stimulation (DBS), whereas patients not treated with DAs perform comparably to HCs (Djamshidian et al., 2013; Voon, 2017).

Neuroimaging studies indicate that patients with ICDs have altered decision-related processing. They exhibit increased activation of the right middle frontal gyrus during choice commitment, abnormal rewards-outcome sensitivity in the frontal pole, and reduced probability-sensitive cerebellar responses, indicating impairments in rewards prediction and probabilistic reasoning (Ruitenberg et al., 2022). Structural and functional connectivity studies show that these patients have a decreased frontal-basal ganglia coupling as well as increased motor cortical-cerebellar and striatal-cerebellar connectivity, which is associated with higher impulsivity scores and fewer bead draws (Ruitenberg et al., 2018).

Warden et al. (2025) differed between impulsive action and impulsive choice. Impulsive action is defined as the suppression of a rapid, prepotent response, allowing slower cognitive processes to guide behavior, whereas impulsive choice reflects insufficiently considered decision-making that may lead to risky behavior with potential negative outcomes. In PD, studies imply a generalized deficit in impulsive action, with impairments in response inhibition and saccadic control. Impairments in impulsive choice are less generalized. Temporal discounting appears to be largely unaffected, but patients perform worse in evaluating risks and processing negative feedback during decision-making. Overall, impaired impulsive action reported in *de novo* PD patients seems to persist throughout the disease course, even when dopamine medication is discontinued.

### 6.4 Neuropsychiatric symptoms

An increasing body of evidence highlights the significant role of psychiatric and psychological factors in the development and manifestation of ICDs in PD.

#### 6.4.1 Anxiety

Among the psychiatric factors, anxiety has emerged as a consistent predictor of ICD onset (Weintraub et al., 2010, 2013; Ren et al., 2023). In particular, evidence demonstrated that higher levels of anxiety, especially when measured around the time of the PD diagnosis, were significantly associated with an increased likelihood of developing an ICD later in the disease (Waskowiak et al., 2022). These findings suggest that anxiety may act as a predisposing vulnerability factor for ICDs and should be considered a relevant element in risk assessment. A recent study following *de novo* PD patients showed that the strongest predictor for ICDs was the interaction between high trait anxiety and a high dopamine genetic risk score. Individuals who had both elevated anxiety at baseline and greater genetic predisposition for heightened dopaminergic activity showed a significantly higher risk of ICD onset. The authors suggest that genetic vulnerability may contribute to higher trait anxiety, which in turn increases susceptibility to ICDs (Whooley et al., 2025).

Overall, the prevalence of anxiety ranges from 20% to nearly 50% (Heim and Djamshidian, 2025). Similarly, clinically significant depression is present in 35% of PD patients, up to 40.4% in outpatients and 54.3% in inpatients, but with varying severity. Major depressive disorder affects approximately 20% of patients with PD (Prange et al., 2022). Both conditions can often be underdiagnosed, partly due to overlapping symptoms with PD like fatigue or weight fluctuations.

#### 6.4.2 Depression

Depression shows a complex relationship with ICDs, especially when accounting for the use of antidepressants. ICDs are linked to more severe anxiety and depression symptoms regardless of dopamine drug condition, and antidepressants are related to an increased risk of developing an ICD, regardless of affective symptom changes from off to on medication states or DA exposure (Morrow et al., 2023). Studies show that PD patients with ICDs exhibited higher rates of both severe and subthreshold depression compared to patients without ICDs (Santos-García et al., 2021). Certain ICD subtypes, including compulsive eating, excessive gambling, and hobbyism-punding, were associated with elevated depressive symptoms. The presence of an ICD nearly doubled the likelihood of depressive symptoms. Importantly, these associations persisted even after controlling for confounding factors such as antidepressant use and perceived quality of life (Baagil et al., 2023). A longitudinal observational study in Spain found that PD patients with ICDs experienced higher rates of depression and reported poorer mood perception compared to control subjects (Jesús et al., 2020). Similarly, individuals with gambling disorder or other ICDs exhibited poorer mental health prior to diagnosis, with continued decline following onset, suggesting that compromised mental health may act as both a risk factor and a comorbidity of ICDs (Wolfschlag et al., 2025). Notably, other studies found that baseline depression did not significantly predict ICD development, highlighting the complex role of depression in this context (Waskowiak et al., 2022).

#### 6.4.3 Apathy

Apathy has also been associated with PD and different ICD subtypes (Antonini et al., 2017; Baig et al., 2019). Apathy is described as a loss of motivation resulting in decreased goal-directed actions, and it is a complex condition with cognitive, affective, and behavioral components (Béreau et al., 2023). Clinically, the patients exhibit little initiative, emotional bluntness, a lack of interest in their environment, and reduced participation in social activities (Heim and Djamshidian, 2025). Although apathy can occur alone, it commonly coexists with other mood disorders, such as depression, anxiety, or ICDs (Béreau et al., 2023). PD patients with both ICDs and apathy exhibit more severe disease progression, with higher Hoehn and Yahr stages, and more pronounced non-motor symptoms compared to those with only ICDs, only apathy, or neither (Maggi et al., 2024). Whereas depression was more closely linked to compulsive shopping, apathy was identified as a risk factor for binge eating and the occurrence of multiple ICDs (Ren et al., 2023). Additionally, impaired executive control over time was specifically associated with compulsive buying in PD patients exhibiting both ICDs and apathy (Maggi et al., 2024). These findings indicate that apathy may contribute to more severe ICD phenotypes and influence specific compulsive behaviors.

#### 6.4.4 Mood dysregulation

Mood dysregulations, such as (hypo)mania, irritability, and aggression, are closely linked to ICDs in PD, suggesting a wider impact of dopaminergic treatment on limbic and motivational circuitry. Non-motor fluctuations show a distinct alternation as hyperactivity and hypomania are present in ON/hyperdopaminergic phases, whereas depressed mood, anxiety, and apathy are more dominant in OFF times. Studies therefore suggest that mood swings correspond to changes in dopaminergic tone (Rieu et al., 2016). Hypomania, or manic affective syndromes, are well-described in the context of dopamine replacement therapy (DRT), particularly in DDS. The patients escalate medication use, and experience euphoria, dysphoria, or dysphoric crash states when doses wear off (Wu et al., 2012; Weintraub and Mamikonyan, 2019). Medication-induced hypomania is also common in patients with pathological gambling, who tend to have more impulsive sensation-seeking tendencies and earlier PD onset (Evans et al., 2009).

Along with mood elevations, ICDs and DDS frequently involve increased irritability, disinhibition, low frustration tolerance, and poor insight. Periods of not being able to experience rewarding behaviors or access medication can cause anxiety, depression, or irritability (Pontone et al., 2006; Ceravolo et al., 2010; Okai et al., 2011; Carbone and Djamshidian, 2024). Aggressive behavior can also develop, ranging from verbal aggression and paranoid reactions to impulsive aggression during medication-seeking, or sexual demands. During phases of excessive medication use restlessness and akathisia, as well as hypomanic behavior can also occur (Stamey and Jankovic, 2008; Evans et al., 2009).

### 6.5 Effects of medication on behavior

Evidence suggests that PD patients have significant deficits in response inhibition across tasks such as the Stop-Signal Task, Go-NoGo, Stroop, and anti-saccade paradigms. A meta- analysis showed that medication seems to improve task performance in the early stages, while it may deteriorate inhibitory control in the more advanced stage. These results support the overdose hypothesis and its related neuroplastic changes due to chronic dopaminergic changes (Cools et al., 2003; Manza et al., 2017). However, other studies show that task performance depends on dopaminergic status (on versus off), but also on illness duration. In a retrospective analysis of patients with DDS, about half of the patients remained symptomatic until death despite gradual reduction of dopaminergic therapy. Interestingly, those who remained symptomatic received higher maximum dosages of DA (Barbosa et al., 2018). PD groups with DA treatment gathered significantly less information and made more irrational decisions in the Beads Task than non-DA patients, who performed comparably to controls. Deep brain stimulation had no effect (Djamshidian et al., 2013). In early PD, patients in medicated states (PD-ON) perform more similarly to controls, whereas in later disease stages, medication is associated with more severe inhibitory impairments (Manza et al., 2017).

Furthermore, dopaminergic medications can affect rewards and punishment processing, impulsivity, and decision-making. For instance, PD-ON patients are more sensitive to rewards than punishments, but PD patients off-medication (PD-OFF) learn more from punishments (Rutledge et al., 2009; Balasubramani et al., 2015). DA have been shown to reduce punishment-based learning by decreasing orbitofrontal cortex responses to negative prediction errors (Van Eimeren et al., 2009), as well as to enhance striatal and orbitofrontal activation during rewards learning in patients with pathological gambling or compulsive shopping (Voon et al., 2010). In addition, DA can increase risk-taking behavior and decision-making speed without improving inhibitory control, indicating a type of reflection impulsivity (Voon et al., 2010; Napier et al., 2015). PD patients in the levodopa on state exhibited a longer decision time than HCs (Kobayashi et al., 2019). Additionally, PD-ON patients performed significantly worse on the IGT than healthy controls. However, short-term medication withdrawal did not restore performance, as PD-OFF patients off also showed impairments relative to controls (Evens et al., 2016).

## 7 Management of ICDs

Management of ICDs is complex and often challenging for patients, their family members and treating physicians, often due to lack of insight. Therefore, prevention is essential, and patients and their families should be informed of potential personality changes that may occur during therapy over time. Furthermore, younger male patients with a personal or familial history of addictive habits, as well as patients with more motor and non-motor symptoms, should be made aware that they are more likely to develop ICDs (Heim and Djamshidian, 2025). If an ICD has been detected, timely intervention it necessary. This requires DA reduction and the screening for other behavioral abnormalities that may indicate developing dysregulation (Napier et al., 2015; Carbone and Djamshidian, 2024). Apart of decrease and cessation of DA, switching to levodopa, or if necessary, increasing the levodopa dose to alleviate motor symptoms, can improve ICD symptoms. However, abrupt discontinuation of DA may lead to dopamine agonist withdrawal syndrome (DAWS), and worsening motor and non-motor symptoms (Rektorova, 2019; Terruzzi et al., 2025). Hospital admission may be necessary.

Although DA discontinuation can result in symptom relief in approximately 40%–80% of patients, neuropsychiatric symptoms often persist for weeks after withdrawal (Carbone and Djamshidian, 2024). Addressing neuropsychiatric comorbidities, particularly sleep disorders, is therefore an important component of management. Antidepressants such as trazodone or the alpha-2 adrenoreceptor antagonist mirtazapine should be considered. Excessive daytime sleepiness, poor sleep efficiency, and fragmented sleep are all linked with an increased risk and severity of ICDs (Scullin et al., 2013; Tang et al., 2024).

In patients with additional psychosis, a short-term treatment with clozapine may be necessary. However, regular blood counts are required and therefore represent a major obstacle (Papay et al., 2014; Carbone and Djamshidian, 2024). Non-pharmacological therapies like cognitive-behavioral therapy can provide additional support (Terruzzi et al., 2025).

**FIGURE 2:**
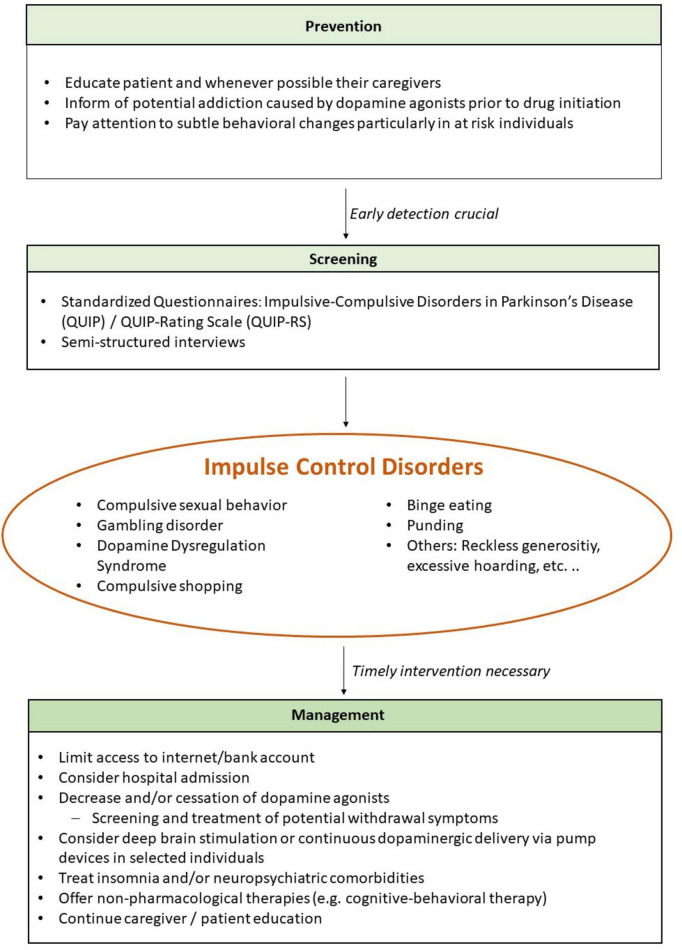
Schematic algorithm for screening/prevention and management of impulse control disorders in Parkinson’s disease.

Recent international expert consensus and guideline efforts provide more explicit recommendations for structured screening, patient–caregiver education, and adjustment of medication. Moreover, this consensus further emphasizes prediction, monitoring, and multidisciplinary care across the disease course (Debove et al., 2024). In this manuscript, we further integrate these clinical updates with neuropsychological vulnerability traits and insight/caregiver considerations to support individualized risk profiling and early detection. A schematic overview of prevention, screening, and management strategies is presented in Figure 2.

### 7.1 Acute vs. chronic effects of medication

Several studies have assessed the effect of on vs. off medication on cognitive performance. Acute dopaminergic medication, including levodopa and DA, modulates neural circuits such as the nucleus accumbens (NAcc), resulting in worse probabilistic reversal learning and altered rewards processing (Voon et al., 2024). Reversal learning is associated with increased NAcc activity only when patients are “off” medication, but dopaminergic replacement impairs this process by altering signaling in the orbital fronto-striatal loop (Poletti and Bonuccelli, 2013). These acute changes in dopaminergic levels can lead to impulsive decision-making in vulnerable patients (Hiebert et al., 2014; Martín-Rodríguez et al., 2025). Notably, even patients who are off short-term medication still performed worse than HCs on the IGT, showing that changes in decision-making networks continue even in off-state (Evens et al., 2016).

Generally, dopaminergic medication reduces both emotional and social bias across affective decision-making tasks, with the strongest effects observed in patients with ICDs. When medicated, the patients show less sensitivity to emotional feedback and social cues than when off medication. They furthermore demonstrate improved learning from negative rather than positive feedback, reversing the off-medication pattern (Djamshidian et al., 2012a). Medication has also been shown to weaken the impact of expectations on subjective emotional states during risky decision-making. Although ICD patients gambled at similar rates regardless the medication status, when off medication, they were less influenced by the expected values compared to PD controls. This suggests that expectations about risky decisions may be decoupled from subjective feelings only in patients with ICDs (Liebenow et al., 2024).

The effects of dopaminergic treatment on cognitive functions also appears to depend on baseline performance (Cools and D’Esposito, 2011). Patients with relatively intact inhibitory control off medication tend to perform worse when treated with DA, which is consistent with a medication-induced “overdose” of relatively intact fronto-striatal circuits. Conversely, ICD patients with poorer baseline inhibition may improve on DA (Wylie et al., 2012).

Additionally, dopaminergic medication can also modulate attention and working memory. Levodopa improves attention and working-memory performance in patients with greater right-hemisphere dopamine deficiency (Hanna-Pladdy et al., 2015). However, effects vary by task and drug. Apomorphine worsens, whereas levodopa improves visual–spatial and visual–object working-memory performance (Poletti and Bonuccelli, 2013).

ON vs. OFF state comparisons show that, while acute dopaminergic medication can increase impulsive tendencies, withdrawal does not completely reverse these effects. The chronic effects, which may also lead to neuroplastic changes, are far more relevant for patients than the acute effects of medication. Therefore, tests comparing the “on” versus “off” medication state provide only a limited explanation.

Long-term consequences can provide a more informative perspective. Deep brain stimulation of the subthalamic nucleus (STN) is associated with mixed effects on ICDs, with several studies indicating reductions improvements. This is largely linked to decreasing the dopaminergic therapies after surgery (Abbes et al., 2018). However, transitory ICD may still occur after surgery. The long-term cognitive and behavioral effects of prolonged dopaminergic treatment remain uncertain. This can occur even at conventional doses (Antonini and Cilia, 2009). Chronic dopaminergic overstimulation, particularly in the ventral striatum and orbitofrontal networks, can result in dysfunctional rewards processing, impaired negative feedback learning, and compulsive behaviors (Weintraub et al., 2010; Poletti and Bonuccelli, 2013).

### 7.2 Continuous dopaminergic therapies

Continuous dopaminergic delivery, also referred to as pump therapies, include levodopa-carbidopa intestinal gel (LCIG) and apomorphine infusion. They aim to reduce motor fluctuations and non-motor symptoms in advanced PD by stabilizing plasma levodopa levels and bypassing gastric emptying (Wirdefeldt et al., 2016).

The emergence of ICDs under LCIG therapy has been evaluated in several studies, with mixed findings. One study reported that 27% of patients developed ICDs, like punding and pathological gambling, during LCIG treatment (Chang et al., 2016). Notably, all affected patients had a prior psychiatric history, and some had pre-existing ICDs before starting the therapy. Other cohorts have reported lower ICD emergence (∼8%). Importantly, LCIG did not trigger ICD recurrence in several patients with prior ICDs (Blaise et al., 2020). An observational study reported that six of eight LCIG-treated patients with ICD had recovered of the behaviors (Todorova et al., 2015). In a multicenter, prospective 6-month study, LCIG treatment resulted in progressive and significant decreases in ICD symptoms, including a 64.4% reduction in QUIP-RS scores. These effects were reported in PD patients with mild to moderate neuropsychiatric symptoms, emphasizing the potential usefulness of LCIG in ICD management (Catalan et al., 2018). The long-term GREENFIELD study found significant improvements in QUIP-RS scores for sexual behavior, dietary habits, and medication use compared to baseline (Lopiano et al., 2019).

Apomorphine, a dopamine agonist, appears to be associated with a lower incidence of newly emergent ICDs (Carbone et al., 2019). Most patients receiving continuous apomorphine infusion did not develop ICDs, and some even improved if previously present. A French national cohort study showed a significant reduction in both ICD scores, and the proportion of advanced PD patients with multiple ICDs after the introduction of subcutaneous apomorphine infusion (CSAI) over a 12–60-month follow-up period. They demonstrated that CSAI treatment is associated with an ICD reduction, when compared to a matched orally treated group (Desjardins et al., 2025). The findings suggest that apomorphine infusion is not linked to ICDs. Additional findings confirm that the majority of individuals receiving apomorphine infusions do not develop ICDs (Barbosa et al., 2022).

Beyond ICDs, infusion therapies have shown to improve non-motor symptoms and the quality of life. LCIG has demonstrated significant and sustained improvements in anxiety, pain, and constipation across different treatment durations (Fasano et al., 2023). Similarly, improvements in mood, cognition, sleep, gastrointestinal and urinary symptoms were observed (Krüger et al., 2017). Long-term studies indicate substantial improvements in motor fluctuations, activities of daily living, and quality of life (Lopiano et al., 2019).

### 7.3 Deep brain stimulation

The efficacy of deep brain stimulation (DBS) in reducing motor impairment in PD is well-established (Fasano et al., 2012). However, its effect on behavioral outcomes has yielded mixed results.

In selected patients without any contraindication for surgery, improvement or remission of ICDs have been reported (Lhommée et al., 2012). At the same time, *de novo* onset of addictive behaviors has also been observed, likely due to electrode misplacement or insufficient reduction of dopaminergic medication, particularly DA (Cossu et al., 2018; Carbone and Djamshidian, 2024). A longitudinal study over 12 months showed no significant longterm difference in ICD severity between DBS and non-surgical patients, with early postoperative reductions in QUIP-RS largely explained by lower dopaminergic medication rather than stimulation effects itself (Hernandez-Con et al., 2023). Other research demonstrates that chronic STN-DBS can alter decision making by decreasing decision time, which is usually seen in patients with ICDs. However, ICD symptoms did not worsen and sometimes even showed mild improvement after surgical intervention (Amstutz et al., 2025). According to computational models, this discrepancy may be explained by the fact that STN- DBS predominantly influences motor or response impulsivity, thereby accelerating decision-making. Unlike DBS, dopaminergic medication mainly influences the rewards-based and risk-sensitive processes, which lead to clinically evident ICDs (Herz et al., 2024).

STN-DBS has consistently been associated with a reduction in ICDs in PD patients, largely caused by the accompanying reduction of dopaminergic medication. Neurobiological models propose that the STN adjusts decision thresholds in situations involving reinforcement or conflict (Frank et al., 2007). Dopaminergic medicine can disrupt learning from negative outcomes by tonically increasing dopamine levels and overstimulating D2 receptors and thereby diminishing conflict-induced slowdown. In contrast, STN-DBS accelerates high-conflict decisions without effecting reinforcement-learning biases, suggesting insufficient regulation of the decision threshold. Computational modeling supported these effects, implying that dopamine-dependent reinforcement learning differs from STN-mediated decision threshold adjustment. This process may explain impulsive actions seen in PD (Frank et al., 2007).

Clinical data also shows mostly positive behavioral outcomes after DBS. Over the course of 2 years, subthalamic stimulation significantly improved both ON-drug euphoria and OFF-drug dysphoria (Lhommée et al., 2018). In a retrospective cohort of 137 individuals, 73% experienced ICD improvement with DBS and medication changes, while 14% developed *de novo* ICDs and 13.5% had persisting symptoms (Healy et al., 2022). Similarly, in a study of 150 patients with PD, the prevalence of ICDs decreased from 17.3% to 12.7% over 4 years, with significant improvements in hypersexuality, gambling, and DDS (Merola et al., 2017). A meta-analysis also found that QUIP-RS scores improved after the operation (Razmkon et al., 2021). Factors associated with a positive outcome are greater reduction of DA or levodopa equivalent daily dose (LEDD), and greater improvement in motor score (Kasemsuk et al., 2017). Acute STN stimulation can reduce risk-taking during gambling activities, potentially due to decreased evidence accumulation during conflict processing (Voon et al., 2017). However, the effects on various impulsivity characteristics vary depending on the stimulation site: ventral stimulation may impair inhibitory control, whereas dorsal stimulation may increase it (Scherrer et al., 2020).

Despite reduction of dopaminergic therapy, *de novo* ICDs might still develop postoperatively in some patients, indicating mechanisms that go beyond medication (Amami et al., 2015; Kasemsuk et al., 2017). Younger age, male gender, higher dopaminergic dosages, and specific personality traits have been identified as risk factors for persistent or new-onset ICDs (Kasemsuk et al., 2017; Merola et al., 2017). Patients with less severe motor symptoms may experience significant ICD improvement but are more likely to suffer postoperative apathy (Santin et al., 2021). Transient psychiatric symptoms are generally common in the initial post-operative and programming phases of DBS (Abbes et al., 2018). Additionally, STN-DBS can also trigger transient mania and worsen behaviors such as pathological gambling, hypersexuality, and compulsive medication use (Romito et al., 2002). It remains unclear whether these effects result from stimulation of the limbic STN or from lesion effects related to surgery.

Overall, DBS is associated with clinical improvement in ICDs in the majority of patients, however, personal risk profiles must be considered for effective management. Additionally, technical and surgical DBS complications should be taken into account as possible contributing factors, as misplaced stimulation can occur which may not be anticipated by the patient or physician (Shotbolt et al., 2012; Rossi et al., 2018). Postoperative adjustment of dopaminergic medication is also critical. Current strategies rely mainly on clinical judgment and patient response, which has limitations. Improper timing, dosage, or reducing medication too early or inappropriately can lead to DDS, psychiatric symptoms, depression, or mania (Diao et al., 2023).

### 7.4 Sleep disturbances

Sleep disturbances are highly prevalent in PD, with the prevalence of nocturnal symptoms and sleep disorders significantly higher than in HCs. This includes disorders like insomnia, restless legs syndrome (RLS), EDS, and RBD (Dhawan et al., 2006; Santos-García et al., 2021). Disturbed sleep perception has additionally been linked to anxiety and depression in PD patients (O’Sullivan et al., 2011). Current research indicates that ICDs in PD are closely connected with sleep dysfunction, as studies using the Parkinson Disease Sleep Scale (PDSS) show that PD patients with ICDs have poorer sleep quality, and higher rates of RLS and EDS (Figorilli et al., 2018). Disordered sleep also appears to reflect more severe depressive symptoms (Antonini et al., 2017). Further studies confirm that impulsivity in PD is associated with EDS and sleep fragmentation (Scullin et al., 2013). RBD may be an additional risk modifier. PD patients with RBD have over double the probability of developing ICD symptoms, and a fourfold risk for pathological gambling, even after accounting for dopaminergic treatment (Fantini et al., 2013).

Some studies link sudden-onset sleep episodes to DA and other dopaminergic therapies (Dhawan et al., 2006). Moreover, impulsive personality traits, a risk factor for ICDs, have also been linked to sleep disturbances (Figorilli et al., 2018). Such sleep disturbances and addictive behaviors may interact bidirectionally, possibly through effects on clock genes that regulate the sleep-wake cycle. Although the causes are unclear, studies suggests that sleep deprivation affects prefrontal-limbic pathways, which then reduce top-down inhibitory control and increase impulsive behaviors. This pattern can be seen in healthy people too (Djamshidian et al., 2015).

## 8 Unresolved controversies

Despite increasing recognition of ICDs in PD several important controversies remain. Reported prevalence estimates vary widely across cohorts (Cossu et al., 2018; Weintraub et al., 2022) likely reflecting heterogeneity in screening tools, cultural factors, disease stage, DA use and exposure complicating direct comparisons across studies. The evidence for a distinct neuropsychological “ICD phenotype” remains also unclear: while global screening tests often show minimal differences, more detailed paradigms suggest task dependent abnormalities (e.g., reflection impulsivity, risk processing, and inhibitory control). Although not fully clear these differences may differ across ICD subtypes (Warden et al., 2025). While the contribution of DA therapy in developing addictions in PD has been well-established other effects of dopaminergic therapy remain opaque. For example the acute “on/off” medication trial designs may not reliably predict persistent behaviors, and thus effects of chronic treatment-related neuroadaptations may be clinically more relevant. Moreover, while the dopamine overdose framework is supported, the causal roles of non-dopaminergic systems (e.g., serotonergic and noradrenergic contributions) remain poorly understood (Vriend, 2018). Finally, treatment evidence is still limited by the paucity of large and subtype-specific trials. Future research should prioritize longitudinal designs and disentangle subtype-specific mechanisms.

## 9 Long-term outcomes

Studies report no significant association between ICD status and longitudinal changes in MMSE scores across domains such as executive function, verbal memory, visuospatial skills, and attention (Erga et al., 2020). Similarly, data from the Parkinson’s Progression Markers Initiative (PPMI) indicate that ICDs are not linked to an increased risk of global cognitive decline or conversion to MCI over time (Meng et al., 2023).

Emerging evidence, however, suggests that ICDs in PD patients may reflect cognitive resilience rather than vulnerability. For instance, a prospective study found that ICD presence at baseline predicted a more favorable cognitive trajectory, with fewer ICD patients exhibiting decline at follow-up compared to controls (Wirth et al., 2024). Consistent with this, Xu et al. (2025) observed that PD patients with ICDs experienced longer survival without conversion to MCI relative to those without neuropsychiatric symptoms. Neuroplastic adaptations in regions such as the parahippocampal gyrus and inferior cingulate cortex have been proposed as potential protective mechanisms underlying this phenomenon. Nevertheless, ICDs are not cognitively benign and are frequently associated with devastating social consequences.

Taken together, these findings underscore the complexity of ICDs in PD. Increasing our understanding of the cognitive and neuropsychiatric mechanisms underlying ICDs will be critical for developing novel treatment strategies that may reduce behavioral side effects without compromising motor symptoms.

## 10 Integrative summary and conclusion

Vulnerability to PD patients with ICDs is often better captured by construct-based neuropsychological profiles than by broad cognitive screening alone. Longitudinal evidence in early PD suggests that impulse-control behaviors may not be consistently associated with global cognitive dysfunction, underscoring the limits of relying on general cognitive measures as proxies for ICD risk.

Second, ICDs in PD should be regarded as a heterogeneous spectrum rather than a single unitary syndrome. Variation across ICD subtypes, severity, and individual vulnerability likely contributes to apparently mixed findings across studies when behaviors are combined or when task selection differs. Recent synthesis work also highlights the importance of distinguishing impulsive action and impulsive choice domains when interpreting behavioral results (Warden et al., 2025). Neuropsychological evidence suggests that ICDs in Parkinson’s disease result from a combination of maladaptive personality traits, increased impulsivity across various domains, and psychiatric comorbidities, despite mostly intact global cognition. These characteristics represent anomalies in rewards processing and inhibitory control that worsen by dopaminergic therapy, shaping both vulnerability to and expression of ICDs (Warden et al., 2025; Kapsomenakis et al., 2023; Waskowiak et al., 2022.)

Third, while DA therapy remains the strongest pharmacological risk factor, medication exposure alone cannot explain why only a subset of patients develop ICDs. Recent evidence supports moving beyond a strictly dopamine-centric account of ICDs which suggests additional neurochemical systems, including serotonergic markers, may play a key role for the development as well for severity of ICDs in PD (Prange et al., 2025).

In conclusion, ICDs in PD should be understood as a heterogeneous, multidimensional spectrum rather than a single clinical entity. Subtype-sensitive assessment approaches are essential to support individualized screening, risk profiling which ultimately may lead to better intervention methods. Consistent with recent expert consensus, structured screening for ICDs should be implemented routinely in clinical practice and should explicitly incorporate information from carers or family members, given the high likelihood of under-reporting by patients. This individualized assessment may likely improve early detection, refine risk stratification, and ultimately enhance patient outcomes.
